# Hydatid cyst in the teres major muscle and brain

**DOI:** 10.1590/0037-8682-0317-2022

**Published:** 2022-10-24

**Authors:** Sadullah Şimşek

**Affiliations:** 1 Nusaybin State Hospital, Department of Radiology, Mardin, Turkey.

**FIGURE 1:**
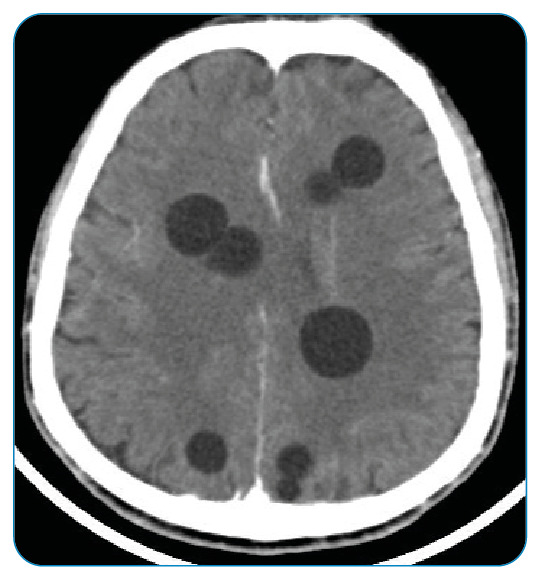
Presence of cystic lesions in both cerebral hemispheres, the largest of which is 2 cm in size, without multivesicular contrast enhancement.

**FIGURE 2:**
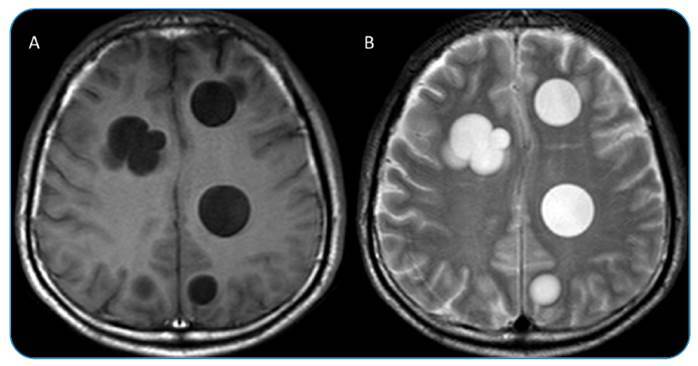
Magnetic resonance imaging of the patient's brain reveals multiple round, multivesicular T1-hypointense **(A)** and T2-hyperintense **(B)** lesions in both cerebral hemispheres.

**FIGURE 3:**
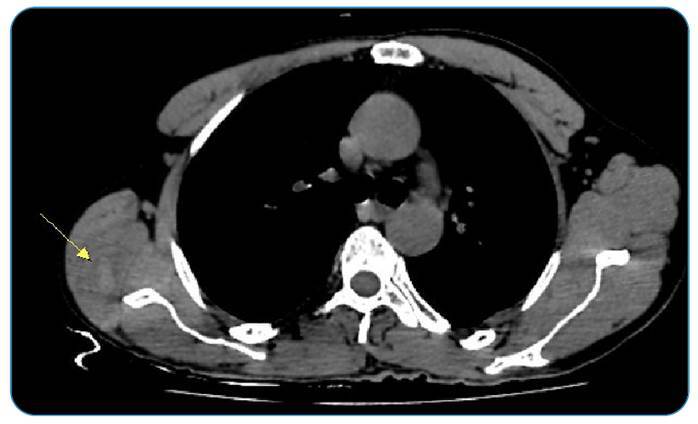
Radiological examination reveals a hyperdense, septate cystic lesion in the right teres major muscle.

A 46-year-old male patient presented with a headache that persisted for 1 year. Physical examination revealed no abnormalities. Contrast-enhanced cranial computed tomography (CT), requested with a provisional diagnosis of a mass, revealed cystic lesions in both cerebral hemispheres, the largest of which was 2 cm in size, without contrast enhancement (Figure 1). Magnetic resonance imaging of the patient's brain revealed multiple round, multivesicular T1-hypointense and T2-hyperintense lesions in both cerebral hemispheres (Figure 2). These findings were significant with respect to hydatid cysts. Non-contrast thoracic CT was performed during the patient's general physical examination. A 22 × 10 mm hyperdense, septate cystic lesion was noted in the right teres major muscle (Figure 3). Postoperatively, a hydatid cyst was confirmed pathologically.

Cystic echinococcosis (hydatid disease) is a zoonotic parasitic disease caused by the ingestion of *Echinococcus granulosus* eggs that can form cysts anywhere in the body. The prevalence of the disease ranges from 0 to 79 cases/100,000 population^1^. Hydatid cysts most commonly affect the liver (55-70%), followed by the lungs (18-35%). The incidence of the cerebral form is 1-2%^2^, and that of the muscular form is approximately 1-5%^3^. Hydatid cysts should be considered in the differential diagnosis of well-defined cystic masses unresponsive to medical treatment in individuals from endemic regions. 
